# Median Arcuate Ligament Syndrome: A Rare Differential Diagnosis of Chronic Abdominal Pain

**DOI:** 10.7759/cureus.84546

**Published:** 2025-05-21

**Authors:** João Victor Bonometti, Pedro Heineberg Muller, Leonardo Werner Rasche, Camila Q Peuckert, Mauricio Jacques Ramos

**Affiliations:** 1 General Surgery, Grupo Hospitalar Conceição, Porto Alegre, BRA; 2 Medicine, Hospital Universitário Canoas, Universidade Luterana do Brasil, Porto Alegre, BRA; 3 Surgical Gastroenterology, Grupo Hospitalar Conceição, Porto Alegre, BRA

**Keywords:** abdominal pain syndrome, celiac trunk compression, chronic abdominal pain, laparoscopic surgery, median arcuate ligament

## Abstract

Median arcuate ligament syndrome (MALS) is a rare condition caused by the compression of the celiac trunk by the median arcuate ligament. It commonly affects young, thin women and presents with chronic abdominal pain, especially postprandial, and weight loss.

We report the case of a 22-year-old female patient with persistent postprandial abdominal pain and significant weight loss. CT angiography revealed compression of the proximal celiac artery. Laparoscopic decompression of the median arcuate ligament was performed with complete symptom resolution and a favorable postoperative outcome.

This case emphasizes the importance of including MALS in the differential diagnosis of chronic abdominal pain in young patients. Early surgical intervention can result in significant symptom improvement and enhanced quality of life.

## Introduction

The median arcuate ligament, a fibrous structure joining the diaphragmatic crura anterior to the aorta, can in some individuals exert pathological compression on the celiac artery, giving rise to a condition known as median arcuate ligament syndrome (MALS) [1]. This vascular compression syndrome is rare and most frequently encountered in young women with low body fat [2]. It typically manifests with nonspecific gastrointestinal symptoms such as upper abdominal pain after meals, early satiety, nausea, and progressive weight loss [3].

Because of the overlap with functional gastrointestinal disorders, the diagnosis is frequently delayed or misattributed. Cross-sectional imaging, especially computed tomography angiography (CTA), has emerged as a vital tool in confirming the characteristic vascular findings of this syndrome [4,5]. Timely diagnosis is important since symptoms often improve significantly after surgical decompression [6].

## Case presentation

A 22-year-old woman was referred for evaluation of persistent epigastric discomfort that had begun one month earlier. The pain was exacerbated by food intake and associated with anorexia, nausea, reduced bowel movements, and a sense of early fullness. She had lost 5 kg unintentionally during this period.

**Figure 1:**
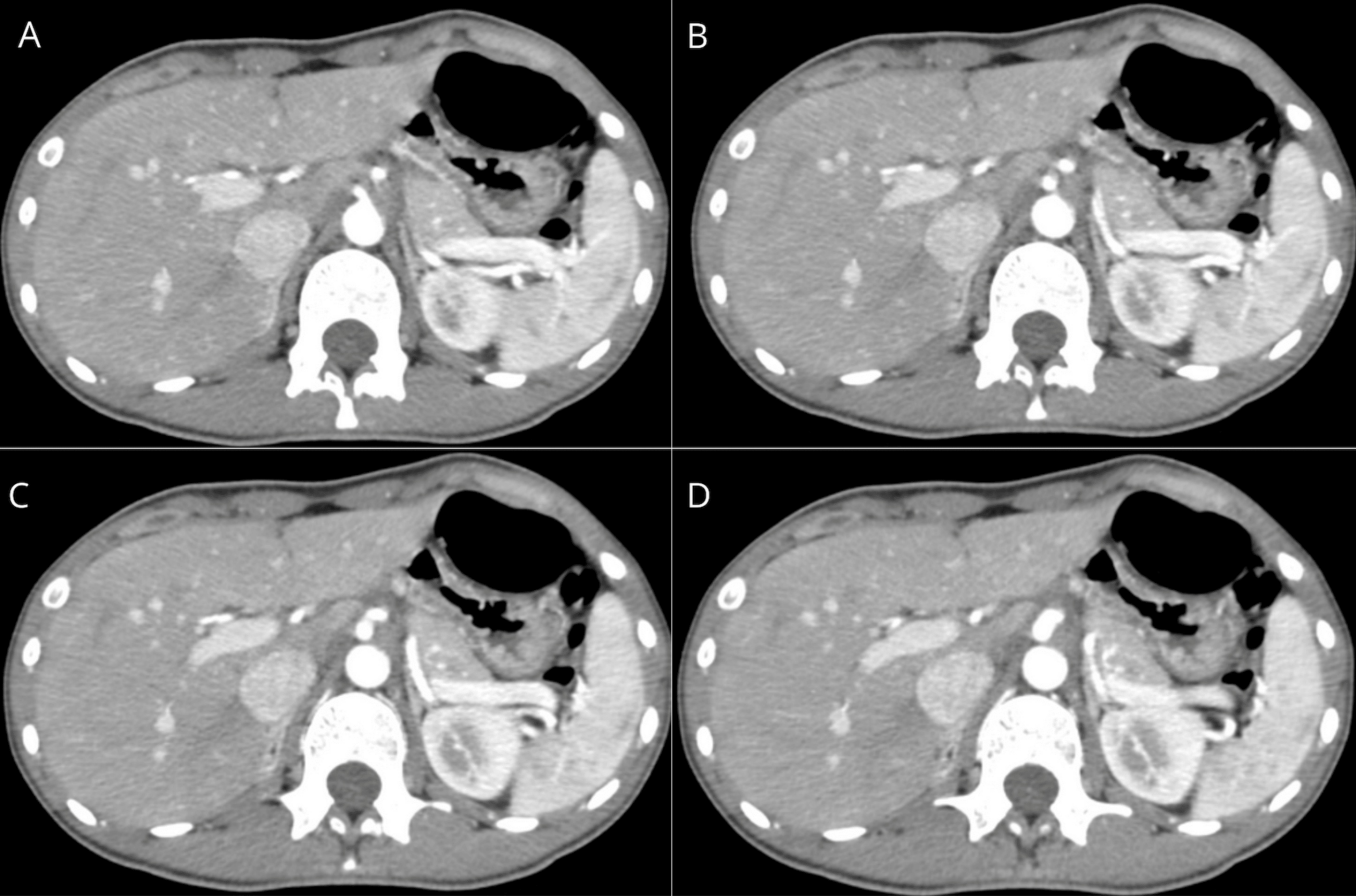
Sequential axial images from CT angiography showing significant luminal narrowing at the celiac trunk origin, with distal re-expansion and visible collateral circulation, suggestive of MALS. MALS: Median arcuate ligament syndrome

Laboratory investigations were within normal parameters initially. Contrast-enhanced CT angiography revealed a distinct focal narrowing at the proximal celiac trunk with a reduction in luminal diameter estimated at approximately 60% (Figure 1). Downstream reexpansion of the vessel was evident, consistent with extrinsic vascular compression. Collateral arterial flow was also observed, raising suspicion for chronic hemodynamic compromise. These findings were consistent with MALS [5,6].

The patient was referred for minimally invasive surgical treatment. Under general anesthesia, a pneumoperitoneum was established using a Veress needle at Palmer’s point. A total of five trocars were inserted: three 10 mm ports (left upper quadrant, left flank, and mesogastrium) and two 5 mm ports (subxiphoid and left lateral). Careful dissection was carried out to expose the celiac trunk. A small adjacent lymph node was removed to facilitate visualization. The fibrous median arcuate ligament was identified and completely divided. Restoration of normal arterial caliber was visualized intraoperatively. The procedure proceeded without complications.

Postoperatively, the patient reported complete resolution of her symptoms and resumed oral intake without difficulty. She was discharged on the third day following surgery. At follow-up, she remained asymptomatic and reported no recurrence of symptoms.

## Discussion

The pathogenesis of MALS is thought to involve both mechanical obstruction of arterial blood flow and irritation of the surrounding sympathetic nerve plexus [2,3]. Despite its rarity, the condition must be considered in young patients with unexplained postprandial pain and weight loss. The symptoms are often vague and can mimic functional gastrointestinal conditions, which contributes to underdiagnosis [4].

CTA is particularly effective for visualizing the focal narrowing of the celiac trunk and its characteristic "hooked" appearance on sagittal views. This appearance is accentuated during expiration, when the diaphragm moves upward, increasing compression [5]. Duplex ultrasound can also aid diagnosis by demonstrating elevated peak systolic velocities in the celiac artery, especially during expiration [4].

Laparoscopic release has become the preferred therapeutic approach due to its reduced morbidity and favorable recovery profile. Complete division of the median arcuate ligament and associated fibrous tissue typically results in decompression of the artery and resolution of symptoms [6,7]. In some cases, neurolysis of the celiac plexus is also performed to address the neurogenic component of pain. Our patient's outcome aligns with literature showing favorable results from laparoscopic intervention in carefully selected patients.

## Conclusions

Although uncommon, MALS is a potentially reversible cause of chronic abdominal symptoms. Clinicians should remain vigilant in evaluating patients with otherwise unexplained postprandial abdominal pain and significant weight loss. High-resolution imaging is essential for diagnosis, and laparoscopic ligament release remains the mainstay of effective treatment.

## References

[1] Loukas M, Pinyard J, Vaid S, Kinsella C, Tariq A, Tubbs RS (2007). Clinical anatomy of celiac artery compression syndrome: a review. Clin Anat.

[2] Kim EN, Lamb K, Relles D, Moudgill N, DiMuzio PJ, Eisenberg JA (2016). Median arcuate ligament syndrome-review of this rare disease. JAMA Surg.

[3] Dunbar JD, Molnar W, Beman FF, Marable SA (1965). Compression of the celiac trunk and abdominal angina. Am J Roentgenol Radium Ther Nucl Med.

[4] Scholbach T (2007). From the nutcracker-phenomenon of the left renal vein to the midline congestion syndrome as a cause of migraine, headache, back and abdominal pain and functional disorders of pelvic organs. Med Hypotheses.

[5] Horton KM, Talamini MA, Fishman EK (2005). Median arcuate ligament syndrome: evaluation with CT angiography. Radiographics.

[6] Rochlin DH, Likes KC, Gilson MM, Christo PJ, Freischlag JA (2012). Management of unresolved, recurrent, and/or contralateral neurogenic symptoms in patients following first rib resection and scalenectomy. J Vasc Surg.

[7] Reilly LM, Ammar AD, Stoney RJ, Ehrenfeld WK (1985). Late results following operative repair for celiac artery compression syndrome. J Vasc Surg.

